# Long-term sperm cryopreservation does not affect post-thaw survival rates

**DOI:** 10.5935/1518-0557.20190066

**Published:** 2020

**Authors:** Juliana R. Pariz, Rosa Alice C. Monteiro, Jorge Hallak

**Affiliations:** 1 Androscience, Science and Innovation Center in Andrology and High-Complex Clinical and Andrology Laboratory. São Paulo, Brazil; 2 Institute for Advanced Studies, University of São Paulo (IEA-USP), São Paulo, Brazil; 3 Section of Andrology - Division of Urology, Faculdade de Medicina da Universidade de São Paulo, São Paulo, Brazil; 4 Reproductive Toxicology Unit - Department of Pathology, Faculdade de Medicina da Universidade de São Paulo, São Paulo, Brazil

**Keywords:** cryopreservation, cryosurvival rates, sperm, sperm bank

## Abstract

**Objective:**

To compare cryosurvival rates of human spermatozoa in a prolonged period of cryopreservation.

**Methods:**

This retrospective study involved 33 cryopreserved semen samples from patients with cancer, between 2002 and 2011. The semen sample was obtained by masturbation and initial semen analysis was performed. The cryoprotectant solution was added and samples were frozen in liquid nitrogen in a slow step-wise process. For thawing, the samples were incubated at 25.0ºC for 15 min, followed by incubation at 36.7ºC for 15 min. The cryosurvival rate (CS) was calculate by CS= [(% total motile sperm post-thaw) x100/(% total motile sperm/tube)]. Each study sample was divided into three aliquots (Study Group; n=23): (I) official patient sample, which was kept cryopreserved for subsequent Assisted Reproduction procedure, cryopreserved between 2002 and 2011; (II) sample destined to post-thaw tests, performed after the sample had been kept cryopreserved for 24 hours; and (III) study sample. Only in 2014, after 3-12 years of cryopreservation, the study samples were thawed and evaluated. To validate the study design, a Validation Group was created including 10 samples obtained between 2014 and 2016, using the same methodology in the study samples. The data was analyzed using the T-test, with a significant *p*-value of 5%.

**Results:**

The mean age was 29.93±9.57 years in the Study Group and 21.80±6.49 years in the Validation Group. No significant difference between the Validation and Study Groups was found in the initial semen analysis (*p*>0.05). After 24 hours of cryopreservation, the cryosurvival rate was 26.11±46.36% in the Study Group and 23.71±57.06% in the Validation Group. Aliquots of the same sample preserved from 3-12 years demonstrated 23.71±57.06% of cryosurvival rate. Thus, no significant difference was found vis-à-vis the cryosurvival rates (*p*=0.56).

**Conclusion:**

We concluded that the method introduced in the late 1990s, which enables the removal of debris, potentially toxic elements and generators of reactive oxygen species from the seminal sample before cryopreservation, exhibited efficiency in maintaining the same cryosurvival rate after an extended period.

## INTRODUCTION

Sperm cryopreservation is the main method for preserving male fertility, which was applied in such situations as artificial insemination with donor sperm, *in vitro* fertilization (IVF) programs and pre-vasectomy micromanipulation of gametes, obtaining sperm by microsurgery, high-risk professions and patients undergoing chemotherapy, radiotherapy and other immunosuppressive therapies (Hallak *et al*., 2000a; Li *et al*., 2010). This process may have several negative effects in sperm, which vary according to the characteristics and/or sample handling.

Damage caused by wide temperature variation, ranging from 36.6ºC to -196.0ºC, such as thermal shock, formation of ice crystals, dehydration and increased intracellular salt concentration caused by osmotic shock (Stanic *et al*., 2000) can impair the structural and functional integrity of spermatozoa via reduced motility and vitality due to mitochondrial damages and in plasma and acrosome membranes (Succu *et al*., 2011), increased DNA fragmentation and generation of reactive oxygen species, lipid peroxidation and apoptosis (Zribi *et al*., 2010; Wang *et al*., 1997; Alvarez & Storey, 1992; Paasch *et al*., 2004). These adverse effects were found in 25 to 75% of spermatozoa undergoing the cryopreservation process (Chian & Quinn, 2010; Hallak *et al*., 1999a).

Mudar para Thus, the improvement of techniques employed in cryopreservation processes, particularly in sample handling and application of diluents and cryoprotectants, which contain osmotically active ingredients, such as glycerol, which is capable of reducing intracellular water and thus reduce the damage caused by the formation of ice crystals. Other components used include glucose, sodium chloride, and human serum albumin, which protect sperm against aggression during laboratory handling, reduce osmotic and oxidative stress, reduce the freezing-point of water and aid in buffering the sample (Chian & Quinn, 2010; Creemers *et al*., 2011). Another important factor for effective cryopreservation is determining the duration of cooling, steam and storage in liquid nitrogen. Similarly, the standardization of thawing steps may enable a satisfactory survival rate of cells, particularly if it is performed following the same steps of freezing process but in reverse (Hallak *et al*., 1999a; 2000b).

 A large number of studies involve sperm cryopreservation using various techniques and species; however, the low recovery rate of viable sperm is still considered unsatisfactory for artificial insemination, required for classic IVF and intracytoplasmic sperm injection (ICSI) procedures (Chan *et al*., 1993; Hallak *et al*., 1998a; 1998b; 1999b; 2000a; 2000b; Vutyavanich *et al*., 2010). In all methods of cryopreservation, the biological activity of sperm can be stored indefinitely in liquid nitrogen, despite the potential morphological reactions occurring at a temperature of -196.0ºC The usual question is how to choose the most appropriate procedure with minimal biological damage, ensuring offspring safety and maintaining the effectiveness of the protocol developed in the laboratory. Each sample is different, even samples obtained from the same patient. Faced with this issue, there is a need for an evaluation to validate the cryopreservation protocol.

Usually, an analysis of sperm survival from a small frozen aliquot is performed 24 hours after freezing to confirm the survival rate of the sample (Hallak *et al*., 1998a), with the goal of providing a preview of how the sample will behave when thawed. Information on sperm viability after years of thawing is scarce in the literature; however, it has been suggested that the default period is 10 years to store sperm, and it has been shown to take up to 55 years in cases of cancer survivors (Sharma, 2011).

In an attempt to analyze the effects of time on the quality of cryopreserved samples, the objective of this study was to compare cryosurvival rates of human spermatozoa in a prolonged period of cryopreservation. In addition, we describe a modified cryopreservation technique adopted in our laboratory, using a slow method, with nitrogen vapor, adding an intermediate step of selecting spermatozoa with better motility and removing elements that are potentially toxic and generators of reactive oxygen species (ROS).

## MATERIALS AND METHODS

In this retrospective study, carried out between 2002 and 2016, thirty-three cryopreserved semen samples (23 study samples and 10 validation samples) were included from reproductive-age patients who chose to donate their samples for scientific research and signed the consent form. This Ethics Committee of the University of São Paulo approved this study (031/2013).

We recruited the patients from private Andrology clinics, with diagnoses of penis and testicle tumors, leukemia, osteosarcoma, adenocarcinoma, and others; and donated samples that were cryopreserved before the start of treatment with chemotherapy and radiotherapy. As for inclusion criteria, we selected patients with diagnosis of cancer that performed initial seminal analysis before the sperm cryopreservation. Exclusion criteria included seminal irregularities in semen evaluation that impaired the post-thawed analysis, such as necrozoospermia, azoospermia and severe oligozoospermia.

Semen sample was obtained by masturbation and prior semen analysis was performed following the current criteria of the World Health Organization (WHO, 1999; 2010). After liquefaction, macroscopic and microscopic parameters were analyzed.

Procedures were carried out to optimize the sample before cryopreservation, such as (i) semen washing, in which debris and cell generators of ROS was removed; (ii) seminal processing by the Isolate^®^ method (Irvine Scientific, Santa Ana, USA), to retrieve viable spermatozoa; or (iii) simple centrifugation to concentrate the spermatozoa in a smaller volume. The semen washing provides the highest yield of spermatozoa and is adequate if semen samples are of good quality; however, with excess cellular debris and round cells. We diluted the seminal sample with modified Human Tubal Fluid^®^ (modified-HTF; Irvine Scientific, Santa Ana, USA) 1:2 to promote removal of seminal plasma and centrifuged at 400 *g* for 15 min. The supernatants were carefully aspirated, and the sperm pellet was resuspended in modified-HTF. The seminal processing by the Isolate method provides the best selection of good-quality spermatozoa, giving good separation from the cell types, because it reduces non-progressive and immotile sperm concentration. We created a 1ml density-gradient medium barrier of 40% (v/v) density-gradient medium over 1 ml of 80% (v/v) density-gradient medium, and 1 ml of seminal sample was added above the density-gradient media. The sample was centrifuged at 400 *g* for 15 min, the supernatants were carefully aspirated and the sperm pellet was resuspended in 1 ml of modified-HTF. A simple washing was performed, and the sperm pellet was resuspended in 1 ml of modified-HTF. In samples with low sperm concentration, we performed a simple centrifugation (400 *g* for 15 min) and removed part of the supernatant, so that the sample became more concentrated in a smaller volume (1 ml).

For sperm cryopreservation, we added the cryoprotectant solution in the 1:1 ratio, at 36.7ºC (Test Yolk Buffer^®^; Scientific Irvine, Santa Ana, USA). Next, the samples were frozen in a slow step-wise process, starting with the equilibration period (8 minutes) at 4ºC, followed by a phase transition (2 hours) in liquid nitrogen vapor at -79ºC and ending with a dip in liquid nitrogen at -196ºC (Sherman, 1986). For thawing, the samples were incubated at 25.0ºC for 15 min, followed by incubation at 36.7ºC for 15 min. The cryosurvival rate (CS) was calculate by CS= [(% total motile sperm post-thaw) x100/(% total motile sperm/tube)] (Henry *et al*., 1993).

Each sample from the Study Group (n=23) was divided into three aliquots: (I) official patient sample, which was kept cryopreserved for subsequent Assisted Reproduction procedures, cryopreserved between 2002 and 2011; (II) sample destined to post-thaw tests, performed after the sample had been kept cryopreserved for 24 hours; and (III) study sample. In 2014, the study samples were thawed and evaluated. To validate the study design we created a Validation Group, consisting of 10 samples obtained in 2014-2016, which were cryopreserved and thawed after 24 hours, using the same methodology of the study samples.

The statistical analysis was performed using the SPSS Statistics 19 software. We analyzed the results using the paired T-test (cryosurvival in the Study Group), and independent T-test (Study *versus* Validation groups) and adopted a significant *p*-value of 5%.

## RESULTS

Table 1 displays the clinical characteristics of the subjects included in study. Their mean age was 29.93 [Standard Deviation (SD) ± 9.57 years old] in the Study Group and 21.80 (SD ±6.49) in the Validation Group. All the subjects were submitted to semen cryopreservation before cancer treatment (chemotherapy and/or radiotherapy).

**Table 1:** Clinical characteristics of the subjects included in the study

Period stored	Samples (n)	Frequency (%)	Clinic diagnosis	Years-old
***1. Study Group***				
12 years	1	4.35	Testis tumor	30
10 years	1	4.35	Pre-Chemotherapy (non-informed tumor)	39
	1	4.35	Testis tumor	37
	1	4.35	Pre-Chemotherapy (non-informed tumor)	17
	1	4.35	Pre-Chemotherapy (non-informed tumor)	39
9 years	1	4.35	Penis tumor	32
	1	4.35	Leukemia	21
	1	4.35	Osteosarcoma	16
	1	4.35	Testis nodule	36
8 years	1	4.35	Sigmoid adenocarcinoma	40
6 years	1	4.35	Pre-Chemotherapy (non-informed tumor)	39
5 years	1	4.35	Pre-Chemotherapy (non-informed tumor)	39
3 years	4	17.38	Mediastinal Seminoma	22
	6	26.08	Pre-Chemotherapy (non-informed tumor)	14
	1	4.35	Testis tumor	28
**Total**	**23**	**100**		**Mean = 29.93±9.57y.o.**
				
***2. Validation Group***				
24 hours	1	10	Leukemia	28
	2	20	Testis tumor	23
				22
	1	10	Sarcoma	16
	1	10	Lymphoma	19
	1	10	Nasopharynges cancer	15
	1	10	Rhabdomyosarcoma	20
	1	10	Adenocarcinoma	37
	1	10	Hypophysis macroadenoma	20
	1	10	Mediastinal tumor	18
**Total**	**10**	**100**		**Mean = 21.80±6.49y.o.**

The initial characteristics of semen samples are depicted in Table 2. No significant difference between the Validation and Study Groups was found concerning sperm concentration (*p*=0.259), progressive total number (*p*=0.120), total motility (*p*=0.589), progressive motility (*p*=0.828), non-progressive motility (*p*=0.264) and non-mobile sperm (*p*=0.840).

**Table 2:** Initial semen characteristics of subjects included in study

	Study group	Validation group	*p* value*
**Sperm concentration (million/mL)**			
Mean ± SD	39.58±26.24	60.86±78.80	0.259
Min-Max	0.3 – 100.0	11.3-298.0	
**Progressive total number (million)**			
Mean ± SD	35.69±29.83	84.22±132.99	0.120
Min-Max	0.1-106.0	1.6-596.0	
**Total motility (%)**			
Mean ± SD	60.90±11.75	63.70±19.71	0.589
Min-Max	30.0-74.0	30.0-95.0	
**Progressive motility (%)**			
Mean ± SD	42.74±15.26	42.10±25.12	0.828
Min-Max	15.0-67.0	0.0-90.0	
**Non-progressive motility (%)**			
Mean ± SD	18.40±9.52	21.60±8.28	0.264
Min-Max	1.0-36.0	5.0-40.0	
**Non-mobile (%)**			
Mean ± SD	35.20±14.05	36.30±19.71	0.840
Min-Max	0.0-70.0	5.0-70.0	

SD: Standard deviation

Min-Max: Minimum and maximum value

* Independent T-test

After 24 hours of cryopreservation, the cryosurvival rate was 26.11±46.36% in the Study Group and 23.71±57.06% in the Validation Group. Aliquots of the same sample preserved for 3 and 12 years demonstrated 23.71±57.06% of cryosurvival rate (Table 3).

The Validation Group had 29% cryosurvival rate in recently cryopreserved samples. Thus, no significant difference was found in sperm cryosurvival rates (*p*=0.56).

**Table 3:** Comparison between total motility and cryosurvival rates in thawing after 24 hours, and thawing after a period of 3-12 years

	Post-thaw test after 24 hours	Post-thaw test after 3-12 years	*p*-value*
**Cryo-survival rate (%)**			
Mean ± SD	26.11±46.36	23.71±57.06	0.892
Min-Max	0.0-68.0	0.0-57.00	

SD: Standard deviation

Min-Max: Minimum and maximum value

* Paired T-test

## DISCUSSION

Sperm cryopreservation process is an important method for gametes preservation, one that maintains cell function and genetic information, enabling future paternity for men with progressive loss of fertility, immunosuppressive treatment or infertility risk (Sharma, 2011). Storage in liquid nitrogen and addition of cryoprotectant substances are standard worldwide procedures for sample maintenance for indefinite amounts of time. The technique's disadvantage is a significant reduction in sperm quality throughout the cryopreservation process, mainly sperm motility, and only 30 to 40% of the sperm remain viable after thawing (Hallak *et al*., 2000a; 2000b; Ranganathan *et al*., 2002); being even less when not cryopreserved properly. Our study was carried out in a specialized andrology laboratory, and the results corroborate literature reports, since our cryosurvival rates in cancer subjects was 30%, regardless of storage period in liquid nitrogen. Calculations for determining the survival rates are based on post-thaw sperm motility, because it is an independent predictor of its functional, metabolic and DNA integrity. Sperm that are motile after thawing are the ones that have survived the stress of freezing and thawing and have proven themselves functionally intact to fertilize oocytes and give rise to a viable embryo (Agarwal *et al*., 1995). Thus, one must consider that even non-motile sperm can be viable, which requires sperm vitality tests.

Establishing effective protocols for human sperm cryopreservation with minimum damage to cells or to improve the sperm quality post-thawing has been discussed and can contribute to Assisted Reproduction techniques. Supplementation of culture medium for freezing with proteins, antioxidants and stimulants to avoid damage to spermatozoa has been widely studied, but the toxicity of these substances, both for the sperm or for the oocyte, remains unclear. The addition of caffeine 2mM (1,3,7-trimethylxanthine; Sigma-Aldrich, USA) in post-thaw samples described by Pariz & Hallak (2016) was associated with increased mitochondrial activity and 38% increase in progressive motility, suggesting that caffeine may be an important tool applied to seminal samples post-thawing. Moreover, the caffeine (2mM) - melatonin (2mM) association seems to preserve mitochondrial activity (Pariz *et al*., 2019). We can also cite the addition of glutamine and pentoxifylline to semen samples, improving motility and fertilization potential (Renard *et al*., 1996; Esteves *et al*., 2007), catalase and ascorbate, which increase motility percentages and reduce ROS production (Li *et al*., 2010).

Despite the fact that cryopreserved samples remain in liquid nitrogen for decades and are viable for fertilization (van Casteren *et al*., 2008), few studies have been devoted to analyze the effects of time storage on sperm quality (Huang *et al*., 2004; Rofeim & Gilbert, 2005). Studies that used frozen-thawed testicular tissue (2-4 years of cryostorage) showed that ICSI, pregnancy and neonatal outcomes are not affected by the cryopreservation duration (Huang *et al*., 2004; Tsai *et al*., 2013). In another study, the authors showed that almost 40% of the patients were able to achieve a healthy live birth with post-thaw semen, and the type of cancer pre-cryopreservation or ART (Agarwal *et al*., 2004) did not affect their chances for success. However, no study has reported cryosurvival or fertilization rates from cryopreserved samples for 10 years or more.

Knowledge of pre-cryopreservation and post-thaw sperm quality plays a role in public health policies and can be used to educate patients who initiate cancer treatment and wish future paternity. Side effects of cancer treatment, such as gonad toxicity and testicular dysfunction, have great potential for affecting fertility, about 25% chance of recovering failure (Moss *et al*., 2016; Williams, 2013). Thus, oncologists, surgeons, and physicians involved in cancer therapy should discuss options available to store spermatozoa before treatment, because each cryopreserved sample is an opportunity to reach a future gestation. The chances of pregnancy depends on the individual quality of each sample, as well as their initial functional characteristics.

Therefore, post -thaw tests are important to predict pregnancy probabilities during the cryopreservation period and to determine the amount of collection/samples to be stored. In the present study, sperm motility was evaluated (% cryosurvival) 24 hours after freezing and compared with samples thawed 3-12 years before. Regardless of storage period in liquid nitrogen, these samples have similar cryosurvival rates. In addition, samples cryopreserved and thawed in 2014-2016 confirmed that the cryosurvival rate was not affected by the storage period. Finally, we were able to validate the consistency and reproducibility of the cryopreservation protocol adopted in our laboratory, because no changes were found in the rates of cryosurvival over the years 2002 and 2016. Thus, it is possible to advise for fertility preservation in liquid nitrogen to patients desiring future pregnancies, who were mainly young males with family plans for more than 12 years.

It is important to report that the number of samples included in this study was a limitation, since authorization for storing samples for study purposes was required. In addition, only samples from cancer subjects were included, and this may be a determining factor for cryosurvival rate having been around 25%. Other metabolic and reproductive diseases or sperm functional characteristics may influence the rate of sperm recovery.

## CONCLUSION

We conclude that the method introduced in the late 1990s showed no difference in rates of cryosurvival over the years. The protocol used in our laboratory, which includes removal of debris, potentially toxic elements and generators of ROS from the seminal sample before cryopreservation was efficient in maintaining the rate of cryosurvival after an extended period. In addition, this technique also presented high reproducibility because the technique applied to Assisted Reproduction was performed according to sperm quality after thawing, which enabled the protocol to be adopted by our laboratory for validation.
